# The Effects of Trained Innate Immunity on T Cell Responses; Clinical Implications and Knowledge Gaps for Future Research

**DOI:** 10.3389/fimmu.2021.706583

**Published:** 2021-08-19

**Authors:** Dearbhla M. Murphy, Kingston H. G. Mills, Sharee A. Basdeo

**Affiliations:** ^1^Human and Translational Immunology Group, Department of Clinical Medicine, Trinity Translational Medicine Institute, St James’s Hospital, Trinity College Dublin, The University of Dublin, Dublin, Ireland; ^2^Immune Regulation Research Group, School of Biochemistry and Immunology, Trinity Biomedical Sciences Institute, Trinity College Dublin, The University of Dublin, Dublin, Ireland

**Keywords:** trained immunity, T cells, adaptive, innate, BCG, beta-glucan

## Abstract

The burgeoning field of innate immune training, also called trained immunity, has given immunologists new insights into the role of innate responses in protection against infection and in modulating inflammation. Moreover, it has led to a paradigm shift in the way we think about immune memory and the interplay between innate and adaptive immune systems in conferring immunity against pathogens. Trained immunity is the term used to describe the medium-term epigenetic and metabolic reprogramming of innate immune cells in peripheral tissues or in the bone marrow stem cell niche. It is elicited by an initial challenge, followed by a significant period of rest that results in an altered response to a subsequent, unrelated challenge. Trained immunity can be associated with increased production of proinflammatory mediators, such as IL-1β, TNF and IL-6, and increased expression of markers on innate immune cells associated with antigen presentation to T cells. The microenvironment created by trained innate immune cells during the secondary challenge may have profound effects on T cell responses, such as altering the differentiation, polarisation and function of T cell subtypes, including Th17 cells. In addition, the Th1 cytokine IFN-γ plays a critical role in establishing trained immunity. In this review, we discuss the evidence that trained immunity impacts on or can be impacted by T cells. Understanding the interplay between innate immune training and how it effects adaptive immunity will give insights into how this phenomenon may affect the development or progression of disease and how it could be exploited for therapeutic interventions or to enhance vaccine efficacy.

## Introduction

In their seminal paper in 2011, Netea and colleagues proposed the idea of trained immunity (also called innate memory or innate immune training) (1) which directly challenged the dogma that only the adaptive immune system had the capacity for immune memory in humans. Trained immunity in mammals is an epigenetic and metabolic reprogramming of innate immune cells, which can occur locally in tissues and in the stem cell niche of the bone marrow, causing altered innate immune responses (2–4). Trained immunity is induced by a primary insult, where innate immune cells are activated and then return to homeostatic function during a period of rest, but maintain the epigenetic and metabolic reprogramming caused by the primary insult. This reprogramming results in an altered immune response to a subsequent, usually unrelated, insult (2–4). It has been reported that β-glucan (a fungal cell wall component), lipopolysaccharide (LPS), the bacillis Clamette-Guérin (BCG) vaccine, adenoviruses and secretory products from helminths can act as the primary stimulus to elicit trained immunity (5–7) (**Table 1**). In addition, there is evidence that endogenous compounds such as oxidised low density lipoprotein (ox-LDL) can also induce trained immunity (17) (**Table 1**).

**Table 1:** Summary of the type of trained immunity induced by different stimuli, their proposed effect on the T cell response and clinical associations.

Ligand	Training Type	Cytokines	Proposed effect on T Cell Response	Effect of T Cell on the Induction of Trained Immunity	Clinical Associations	Ref
BCG	Pro-inflammatory	↑IL-1β	↑Th1	T cells downregulate trained immunity	Protection against infection	(5)
		↑TNF	↑Th17			(8)
		↑IL-6	↓Tregs			(9)
		↑IL-8				
		↓IL-10				
β-glucan	Pro-inflammatory	↑IL-1β	↑Th1	Unknown	Protection against infection	(6)
		↑TNF	↑Th17			(10)
		↑IL-6	↓Tregs			
		↓IL-10				
FHTE	Anti-inflammatory	↑IL-10	↑Tregs	Unknown	Protection against EAE	(11)
		↑IL-1RA	↓Th1			(12)
		↓TNF	↓Th17			
		↓IL-12p40				
Adenovirus	Pro-inflammatory	↑MIP-2	↑Th17	CD8 T cell IFN-γ required to induce trained immunity	Protection against infection (via enhanced neutrophilia)	(13)
		↑KC				
		↑IL-1β				
LPS	Tolerance	↑IL-10	↑Tregs	Unknown	Sepsis Immunoparalysis	(14)
		↓TNF	↓Th1			(15)
		↓IL-6	↓Th17			(16)
oxLDL	Pro-Inflammatory	↑TNF	↑Th-1	Unknown	Athersclerosis	(17)
		↑IL-6	↑Th-17			
		↑IL-8				
MuHV-4	Anti-inflammatory	↓IL-5				
		↓IL-13	↓Th2	Unknown	Reduced allergic reaction to HDMs	(18)
		↓IL-4	↑Th1			
		↓IL-6				
		↓IL-10				

Trained immunity has been well documented in monocytes but can also occur in differentiated macrophages, dendritic cells (DC) and natural killer (NK) cells (19–23). Since many innate immune cells are short-lived, the functional effects of trained immunity, which can last several weeks (5, 24), and in some cases, up to a year (8, 25), are attributed to immune reprogramming of the haematopoietic stem cells in the bone marrow (10, 24–26). This results in newly generated myeloid cells exhibiting the features of trained immunity when they egress from the bone marrow into the circulation (24, 26).

Innate immune training is a medium-term phenomenon which can mediate non-specific or specific immunity against pathogens. This is distinct from adaptive immune memory, which generates specific long-term memory, in the form of memory T and B cells that clonally expand and rapidly respond upon re-exposure to the same pathogen. The effect of trained immunity on adaptive immune responses has not been thoroughly explored. Trained immunity is likely to impact on the adaptive immune response due to the heightened production of T cell polarizing cytokines and increased expression of markers associated with antigen presentation. In addition to polarising naive T cells, cytokines produced by innate cells also regulate memory T cell function and lineage commitment. Therefore, trained immunity may have an impact on T cell plasticity and may alter the Th1/Th2 or Treg/Th17 balance. In addition, T cell responses may have an upstream effect on trained immunity through activation or modulation of innate immune cells.

Understanding the influence of trained immunity on adaptive immune responses will help us to better understand the impact of trained immunity in the control of infectious diseases or in promoting pathology in immune-mediated diseases. It may also aid the design of more effective vaccine strategies that combine the induction of trained immunity with the generation of traditional adaptive immune memory.

## Evidence for Trained Immunity Impacting T Cell RESPONSES

**Figure 1:**
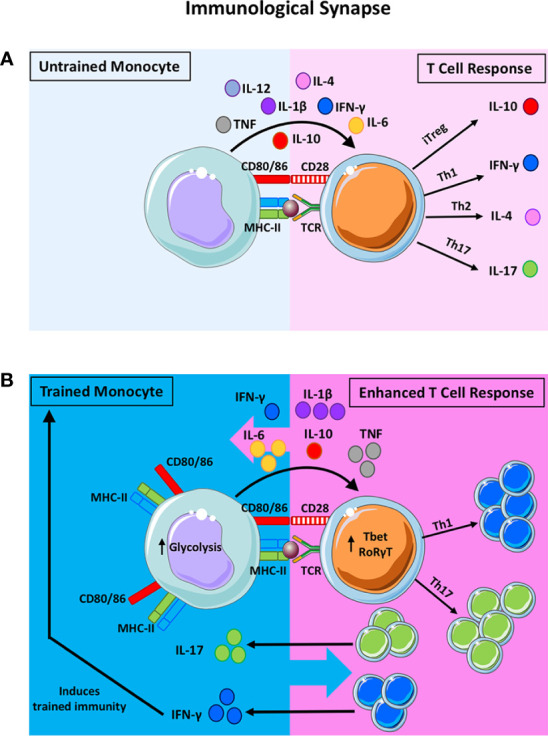
The potential impact of trained immunity on the bidirectional innate-adaptive immune synapse. The traditional synapse between antigen-presenting cells (APC) and naïve T helper cells is depicted in **(A)**. The innate APC processes and presents antigen on the MHC-II molecule to provide the first signal to activate the T cell. Upregulation of co-stimulatory molecules on the APC and their ligation to the cognate receptors on the T cells provide the second signal required for T cell activation. Cytokine production by the innate APC then polarises the naïve T cell into discreet subsets. For example, the presence of IL-10 will induce the formation of T regulatory cells (Treg) whereas IL-12 and IFN-γ will polarise T cells towards a Th1 lineage. IL-4 promotes Th2 polarisation and IL-1β and IL-6 (along with other innate cytokines) promote Th17 development. This synapse is often viewed as unidirectional, however, there is evidence to suggest that T cell cytokines can impact also APC function. In addition to being the critical control point in the polarization of naïve T cells, this synapse is also crucial for reactivating memory T cells in tissues. Under these circumstances, innate cytokine production can alter the functional profile of previously lineage committed T cells. When trained immunity is induced in innate cells, their APC function and cytokine production is markedly enhanced **(B)**. Trained innate cells exhibit increased expression of MHC-II, costimulatory molecules (CD80/CD86) and an upregulation of aerobic glycolysis. Moreover, trained monocytes produce significantly more TNF, IL-1β and IL-6 compared with untrained monocytes. These features of proinflammatory innate immune training suggest that T cell polarization may be skewed in favour of Th17/Th1 responses, as indicated by the expression of the master transcription factors RORγT and Tbet, respectively. In the same manner, lineage committed T cells that are prone to functional plasticity, such as the Th17 and Treg lineages, may be altered by trained immunity and directed down alternative lineage fates. Enhanced IFN-γ and IL-17 production from T cells can impact innate cell function. In fact, IFN-γ production has been shown to induce trained immunity in tissue resident macrophages. Notably, alternative types of trained immunity that result in enhanced IL-10, for example, will alter T cell fate towards Treg cells, which may in turn impact innate APC function. We propose that, trained immunity alters T cell fate and function which may in turn alter innate cell function. Depending on the primary insult that induces trained immunity, the pro-inflammatory versus anti-inflammatory nature of this bi-directional synapse will be altered depending on the cytokine milieu and the cellular functions that are induced by trained immunity.

Monocytes that undergo innate immune training with adenovirus or BCG have enhanced antigen presenting function due to increased expression of MHC-II and the co-stimulatory molecules CD80 and CD86 (13, 27). These two signals are critical to the immune synapse between antigen presenting cells (APC) and T cells (**Figure 1A**). A third signal, cytokine production by the APC, directs the polarisation of naïve T cells into discrete subsets and can alter the function of effector T cells that are already lineage committed. Therefore, altered cytokine production in myeloid cells following trained immunity is likely to impact T cell polarisation and lineage fate (**Figure 1B**). Indeed, Kleinnijenhuis et al., suggested that the increased expression of pathogen recognition receptors and increased production of pro-inflammatory cytokines by innate immune cells contributes to enhanced T cell responses (8). However, there is a paucity of data examining the effects of trained immunity on the polarisation of effector T cell subtypes and Treg cells. Since DC are the key APC in triggering naïve T cell activation, identifying the effects trained immunity on DC maturation and function will be critical to understanding the interplay between trained immunity and adaptive immune responses. Further elucidation of the effects of trained monocytes and macrophages on tissue-resident memory T cell responses will also be beneficial in understanding the impact of trained immunity during a subsequent insult.

Monocytes from BCG-vaccinated adults have increased production of IL-1β, TNF and IL-6 upon restimulation *ex vivo* with unrelated pathogens, *Staphylococcus aureus*, *Candida albicans* and the yellow fever virus vaccine strain (5, 8, 9). IL-1β and IL-6 are key drivers of Th17 cell responses and can promote functional plasticity towards the more pro-inflammatory ex-Th17 cell fate, where Th17 lineage cells produce IFN-γ, which is associated with protection against certain infections (28) but also with immune-mediated pathology (29). This suggests that altered innate cytokine production induced by trained immunity is likely to impact on T cell differentiation and fate. Consistent with this, BCG vaccination is associated with elevated IFN-γ and IL-17 production from PBMC stimulated *ex vivo* with *Mycobacterium tuberculosis* and unrelated pathogens (5) (8) (**Table 1**). Taken together, these findings suggest that vaccination with BCG may enhance Th1 and Th17 cell responses to unrelated stimuli, although the evidence does not definitively show that the IFN-γ and IL-17 are produced by T cells (8). In addition, this study did not mechanistically link the increased innate IL-1β and TNF produced by monocytes in BCG vaccinated individuals to the longer-term effect on Th1 or Th17 cells. We propose that this heterologous Th1 and Th17 immunity generated by the BCG vaccine may, at least in part, be mediated by the altered function of innate cells that have undergone trained immunity. We also hypothesise that trained immunity may promote Th17 cell plasticity towards an ‘ex-Th17’ phenotype (which means that they start producing IFN-γ), therefore, the increased IFN-γ secretion in this system may be produced by both Th1 and Th17 lineage cells.

The precise role that T cells play during the induction of trained immunity is unclear. Yao et al. reported that trained immunity can be induced in tissue resident alveolar macrophages (AM) following infection with a respiratory adenovirus vector *in vivo* in a mouse model (13) (**Table 1**). These ‘trained’ AM exhibited heightened MHC II expression, increased glycolytic metabolism and a “defence-ready” gene signature at 4 and 16 weeks post infection (13). Interestingly, the depletion of T cells completely abrogated the expression of MHC-II, chemokine production and metabolic reprogramming in the AM. Consistent with this, SCID mice infected with the adenoviral vector failed to induce trained immunity in the AM population, however, transfer of IFN-γ-producing CD8 T cells to the airways of the SCID mice restored the phenotype and function of the memory AM (13). By depleting T cells at different timepoints, this study established that T cells were required initially to generate the memory AM, but are not required later on to maintain this cell population (13). In addition, they showed that IFN-γ deficiency diminished trained immunity in the AM. To definitively show that IFN-γ production from the CD8 T cell was required for the induction of trained immunity in the AM, CD8 T cells from wild type mice infected with adenovirus vector were transferred to RAG mice and this rescued the trained immunity phenotype in the AM, whereas CD8 T cells from IFN-γ-defective mice did not alter the AM (13). These findings indicate that IFN-γ production by T cells plays a critical role in establishing trained immunity in tissue resident AM and supports the hypothesis that trained immunity can be influenced by T cell responses. This study did not investigate if these “memory” AM modulated T cell responses in the lung during the subsequent infection with *Streptococcus pneumoniae*. However, when T cells were depleted after the memory AM population were established but before challenge with *S. pneumoniae*, these mice were also protected against the bacterial infection (13). This indicates that the T cell response is not necessary to mediate protection against bacterial infection after induction of trained innate immunity by the adenoviral vector. It is possible that the T cell population may be altered in the lung by the enhanced function of these “trained” AM and this may influence disease outcomes in humans.

Conversely, there is evidence to suggest that T cells may play a role in downregulating trained immunity since monocyte function can be enhanced in the absence of T cells (5, 6, 24). It is plausible that T cells have distinct effects on trained immunity depending on the initial training stimulus, the context of inflammation or at different sites. For example, the presence of T cells in the bone marrow appears to downregulate trained immunity, resulting in monocytes with reduced bactericidal capacity compared with T cell-depleted bone marrow (24). Interestingly, there is a requirement for IFN-γ in reprogramming the haematopoietic stem cells in the bone marrow to elicit trained immunity in cells undergoing myelopoiesis (24), underscoring the prominence of its role in this process but the cellular source of IFN-γ may be innate or adaptive, depending on the context.

### Anti-Inflammatory Trained Immunity

The majority of studies to date describe trained immunity as enhancing pro-inflammatory responses, however, it was recently reported that myeloid cells can be trained towards an anti-inflammatory phenotype following exposure to helminth products (11, 12). Macrophages trained *in vitro* with *Fasciola hepatica* total extract (FHTE; **Table 1**) or *F. hepatica* excretory secretory products (FHES) showed heightened production of the anti-inflammatory cytokines IL-1RA and IL-10, but reduced production of the pro-inflammatory cytokines TNF and IL-12p40 (11). IL-10 induces Treg cells while IL-1RA acts as natural inhibitor of IL-1β signalling, stunting the Th17 response (30, 31). Suppressed IL-12p40 and TNF dampens the Th1 cell response (11, 32). FHES administered to mice *in vivo* altered the hematopoietic stem cell niche in the bone marrow and resulted in hyporesponsive monocytes at different sites in the mice (12). These data also showed that this anti-inflammatory trained immunity induced by FTHE or FHES was associated with reduced Th1 and Th17 responses and attenuated EAE (11, 12), the animal model of autoimmune multiple sclerosis, which is mediated by pathogenic Th1, Th17 and ex-Th17 cells (33).

## Innate Immune Training and Heterologous T Cell Immunity

Many vaccines that are known or postulated to induce trained immunity are also associated with promoting heterologous T cell immunity; a phenomenon whereby T cells can cross-react and promote immunity against unrelated pathogens. Deleterious effects of cross-reactive T cells causing pathology post infection are well documented in humans; for example, dengue virus-induced haemorrhagic fever and Epstein-Barr virus (EBV)-induced mononucleosis (34–36). However, the beneficial effects of heterologous T cell immunity are harder to recognise clinically.

Heterologous T cell immunity can be mediated through T cell receptors that can bind to two or more MHC-peptide complexes, initiating cross-reactive T cell responses (37). Alternatively, memory T cells can also be activated in a bystander manner by cytokines in the absence of antigen specificity (38–41). Cytokines documented to induce this bystander activation of T cells include type 1 and type 2 interferons (38, 39, 42). Moreover, IL-1β or IL-18 can synergise with IL-23 to induce bystander activation and production of IL-17 and IFN-γ from CD4 T cells in the absence of TCR stimulation (43).

A study using an animal model of respiratory syncytial virus (RSV) demonstrated that previous influenza infection protected mice against illness, weight loss and eosinophilia during RSV (44). When the interval between the two infections was extended from 3 weeks to 21 weeks, the protective effects of influenza infection mediated by reduced eosinophilia were maintained. An examination of the possible mechanisms of protection found no evidence for cross-reactive T cells (44). However, there was some evidence to support bystander activation. Influenza virus-specific T cells returned to the lung during RSV infection and produced IFN-γ, however, the resident memory T cells in the lung did not confer the same bystander protection against eosinophilia as the influenza virus-specific T cells. The authors concluded that the shift in the Th1/Th2 cytokine balance caused by the presence of influenza virus-specific T cells is likely to mediate protection, since the influenza infection promoted IFN-γ and decreased IL-4 in the lung (44). In addition, previous influenza infection was associated with reduced TNF and increased IL-10, suggesting the enhanced anti-inflammatory cytokine production could be responsible for protecting the animals against immune-mediated pathology during RSV infection (44). This study indicates that the history of infection has an impact on subsequent control of pathogens, even after a significant period of rest (21 weeks). Trained immunity is likely to play a role in this experimental model of heterologous immunity, however, there is no definitive evidence that innate cell function was altered by the primary infection (44). The exact role of innate and adaptive immune responses in heterologous immunity is still unclear and further research is required to establish a cause-and-effect relationship between trained innate immunity and heterologous T cell immunity.

Heterologous immunity generated by the BCG vaccine is well documented and accounts for its protective effects against all-cause mortality in infants (45, 46). This is thought to be mediated by both trained innate immunity and heterologous T cell immunity. BCG vaccination in humans has been shown to induce heterologous Th1 and Th17 cell responses in response to *S. aureus* and *C. albicans in vitro* (8). Likewise, immunisation with the BCG vaccine in mice, conferred protection against a subsequent vaccinia virus infection, which was mediated by CD4 T cells (47). Since there is ample evidence that BCG induces innate immune training in myeloid cells (5, 48), it is plausible that the training of these cells results in the activation of T cell mediated heterologous immunity. T cell activation thresholds or TCR specificity may be altered in the context of hyperresponsive trained innate cells that exhibit increased cytokine outputs in addition to enhanced APC function. However, definitive evidence that BCG-induce trained immunity is directly associated with heterologous T cell immunity remains elusive.

The vaccinia virus, used as the smallpox vaccine, has been shown to reduce susceptibility to various infectious diseases and to promote healing of chronic skin rashes (49, 50). Innate immune training has been suggested as the mechanism responsible for the non-specific effects of vaccinia virus in humans, with both epidemiological and experimental data linking vaccinia virus to trained immunity (51–55). Vaccinia virus has also been associated with heterologous T cell immunity to HIV since CCR5^+^ T cells from smallpox-vaccinated individuals showed significantly lower HIV-1 replication compared with CCR5^+^ T cells from unvaccinated controls (56). Moreover, HIV replication was markedly reduced in T cells from people who were immunized with vaccina virus 6 months earlier, similar to the effect observed in cells recovered 3 months post vaccination (56). This prolonged effect on virus replication suggests that the mechanism is different to that observed during an active viral co-infection with measles or dengue fever virus, for example, which are known to competitively inhibit HIV replication (57, 58). In a follow-up study that investigated the non-specific effects of vaccination on protection against HIV replication, increased production of IL-8, MIP-1α and MIP-1β were detected in serum of people who had received multiple vaccinations (59). The authors concluded that the persistently elevated chemokines observed several months post vaccination may play a role in reducing viral replication in T cells from vaccinated people compared with unvaccinated controls (56, 59). Taken together, the evidence that vaccina virus induces trained immunity (51), together with the data demonstrating heterologous T cell immunity to HIV, and the associated role of chemokines, suggests that these phenomena may plausibly be linked.

These data suggest that vaccine or infection induced trained immunity and heterologous T cell immunity occur concomitantly and may be integrally linked, however, definitive evidence for this is yet to be established.

## The Clinical Significance of the Link Between Innate Immune Training and T Cells

While the clinical benefit of trained immunity in mediating protection against infection is clear, it is important to note that trained immunity may also induce inflammatory pathology (2, 60), in conditions known to involve the induction of pathogenic T cells. Increasing evidence is emerging in support of this hypothesis, as discussed below, however, a knowledge gap remains as to whether trained immunity affects the induction or propagation of pathological adaptive immune responses in different disease settings.

### Allergy

Food allergy is thought to be a disease of the adaptive immune system, however, there is emerging evidence for dysfunctional innate responses in allergy (61, 62), and a possible role for trained immunity in the development of allergy (63, 64). Infants who are allergic to eggs have increased frequencies of circulating monocytes (which express elevated HLA-DR), reduced numbers of Treg cells and an increased monocyte:CD4 T cell ratio compared with healthy controls (61). Interestingly, T cell-depleted PBMC isolated from egg allergic infants showed significantly increased production of TNF, IL-6, IL-8 and IL-1β in response to LPS stimulation compared with non-allergic controls (61). The increased production of TNF, IL-6, IL-8 and IL-1β by monocytes has been previously associated with a trained immunity phenotype (5, 6, 9, 65). Moreover, the study compared allergic infants that had been subjected to an oral food challenge with those that did not and found no differences in concentrations of IL-6 or TNF in the serum. Consistent with this, the elevated LPS-induced cytokine production by T cell-depleted PBMC from egg-allergic infants were not significantly different on the day of oral food challenge or on a different day. We postulate that this may indicate a role for trained immunity during allergy. Since allergy is mediated by Th2 responses, a mechanistic role for trained immunity in the induction of Th2 cell mediated allergy remains to be determined. Given that the cytokines reported above are more likely to promote Th1 and Th17 responses, further work is required to determine the link, if any, between trained immunity and the induction of allergen-specific T cells. Interestingly, the authors also report significantly diminished IL-12p70 in allergic infants in resting CD3-depleted PBMC and those stimulated with LPS. Therefore, the purported trained immunity that causes increased TNF, IL-6, IL-8 and IL-1β in these infants while concomitantly decreasing IL-12p70 production compared with non-allergic controls may serve to skew the balance in favour of Th2 cells by the limiting Th1 differentiation induced by IL-12p70.

Studies in a murine asthma model have shown that infection with a murine gamma herpes virus (MuHV-4; **Table 1**) 30 days before induction of experimental asthma using house dust mite (HDM) induced a population of regulatory monocytes in lung that inhibited the development of experimental asthma (18). Virally infected mice had lower amounts of the pro-asthmatic innate cytokines IL-5, IL-13, IL-6 and IL-4 in their lungs compared with uninfected control mice (18). Cells isolated from the mediastinal lymph node had reduced proliferative capacity and had reduced production of IL-5, IL-13, IL-4, IL-6 and IL-10 relative to control mice, whereas IFN-γ production from these cells remained unchanged compared with the control, suggesting that the Th2 response, but not Th1 responses, was specifically affected by the infection with the virus (18).

The trained immunity induced by MuHV-4 resulted in reduced production of Th2 polarising cytokines from monocytes, leading to a decrease in Th2 activation and allergic inflammation. This provided strong evidence supporting a role for infection in the prevention of allergy, as postulated in the hygiene hypothesis.

These data suggest a role for trained immunity in influencing the pathogenic T cell responses observed during allergy. However, there is a lack of empirical evidence showing that trained immunity in innate cells modulates T cell responses during allergy.

### Atherosclerosis

Atherosclerosis is a chronic inflammatory disease of the arterial wall, mediated by CD4 T cells that recognise ox-LDL and are commonly found within atherosclerotic plaques (66, 67). Trained immunity has been proposed as an underlying driver of atherosclerosis (17, 68, 69). Ox-LDL enhances pro-inflammatory cytokine production and foam cell formation through the epigenetic reprogramming of monocytes (**Table 1**) (17, 70). While the role of monocytes in the pathogenesis of atherosclerosis has been well established, the mechanism behind persistent vascular inflammation has remained elusive. The long-lasting hyperinflammatory monocytes, which display epigenetic reprogramming following exposure to ox-LDL and increased capacity to produce IL-6, IL-8, TNF and MCP-1 upon restimulation with pathogen associated molecular patterns, provide a plausible mechanism for vascular inflammation. There is also evidence of an association between infection and atherosclerotic cardiovascular disease (ASCVD) (71, 72), and trained immunity is postulated to provide that mechanistic link (73).

Since T cells have an established role in the pathogenesis of atherosclerosis, it is plausible that the emerging role for trained immunity during ASCVD may potentiate pathogenic T cell responses. Conversely, pathogenic T cell responses may also serve to amplify trained immunity in monocytes; for example, IL-17A has been shown to increase the adhesion of monocytes to endothelial cells and increase the gene expression of IL-6 in monocytes during atherosclerosis (74). Direct evidence linking trained immunity to pathological T cell responses during ASCVD remains elusive.

### Rheumatoid Arthritis

The aetiology and pathogenesis of rheumatoid arthritis (RA), like most autoimmune disorders, is complex and remains unclear (75). The roles of pathogenic T cells with a loss of self-tolerance have been extensively studied in RA, but more recently the focus is increasingly on innate immune cells as drivers of this disease (76–80). It has already been proposed that trained immunity may have a role in the induction of RA (60, 81), with evidence that hyperplasia in the RA joint is mediated by epigenetic reprogramming (81).

McGarry and Hanlon et al. reported that CD14^+^ monocytes in the peripheral blood of RA patients are metabolically reprogrammed towards glycolysis (82), a key feature of trained immunity (48, 65). Furthermore, when compared with healthy controls, monocytes from the blood of RA patients produced more TNF, IL-6 and IL-1β in response to LPS stimulation *in vitro* (82). These are key cytokines that are enhanced in cells that have undergone pro-inflammatory innate immune training with BCG or β-glucan (5, 6). The frequency of CD14^+^ monocytes is also increased in the blood of RA patients compared with controls, indicative of enhanced myelopoiesis, which is also strongly associated with trained immunity (10, 24, 82). In the setting of established RA disease, the enhanced inflammatory function of monocytes may not be indicative of trained immunity, but it could also reflect chronic inflammation. To determine if these hyperinflammatory monocytes were a precipitating factor for inappropriate inflammation in people with RA, the study examined monocytes in people who were identified as being at increased risk of developing RA (82). The hyperinflammatory CD14^+^ cell phenotype was present in these individuals at risk of developing RA, suggesting that this trained immunity phenotype may precede clinical signs of disease and may therefore be implicated in pathology (82). This study provides empirical evidence that monocytes in the circulation of people with established RA and in those at risk of developing RA are “primed” towards a hyper-inflammatory and hyper-metabolic state, akin to that observed in monocytes that have undergone innate immune training. These data support the hypothesis that trained immunity may contribute to the pathogenesis of autoimmune inflammation, however; whether or not these hyperinflammatory monocytes promote autoimmune T cells responses remains unclear.

RA is a disease with a strong pathogenic T cell response, with plastic Th17-lineage cells implicated in the propagation of inflammation (29, 83). Since Th17 cell plasticity is directed by innate cytokines, trained immunity may have a key role in promoting pathological ex-Th17 cells. Interestingly both pathological Th17 lineage cells and monocytes displaying the features of trained immunity in RA patients are dependent on STAT3 signalling (82, 83). There is evidence that activated monocytes from the joints of patients with RA specifically promote Th17 cell responses, and this is mediated by elevated production TNF and IL-1β in the RA monocytes (84).

There is emerging evidence to suggest that trained immunity may be an underlying feature that promotes the pathogenic phenotype of the hyperinflammatory myeloid cells found in RA patients, and precedes the clinical diagnosis of RA (82). These innate immune responses may subsequently drive a pathogenic T cell response. We hypothesise that trained immunity may therefore contribute to the initiation of disease and the propagation of inappropriate inflammation during relapse.

Since autoimmune diseases more than double the risk of CVD, we postulate that this may be mediated by systemic trained immunity that precedes clinical onset of disease. However, the initial stimuli that induce trained immunity in this setting are unknown. Infectious agents, such EBV, cytomegalovirus (CMV) and *Escherichia coli* have been linked with the induction of RA, although their precise role remains elusive (75). This echoes the link between infectious burden and ASCVD, with trained immunity postulated as the common mechanism (73).

### Sepsis

Sepsis is one of the leading causes of preventable death (85). There is evidence that CD4^+^ T helper cell responses are impaired during sepsis (86) and persistent failure of T cell activation in sepsis is associated with attenuated IFN-γ-producing CD8^+^ cytotoxic T cells and Th17 cell responses (87).

Interestingly, the absence or reduction cell surface expression of the MHC-II molecule, HLA-DR, on monocytes is a common biomarker used to predict disease outcome (88, 89). Furthermore, low HLA-DR expression has been correlated with an impaired TNF response (89, 90).

LPS or endotoxin tolerance occurs in myeloid cells exposed to LPS, resulting in hypo-responsiveness to a second stimulation (7), and is often used to model sepsis. Endotoxin tolerance, although not considered to be a bona fide type of trained immunity, exhibits marked similarities with trained immunity including induction in the bone marrow and a distinct resting period between the primary and secondary stimulation for its generation (**Table 1**) (3). In addition, LPS-tolerized macrophages exhibit metabolic reprogramming that results in reduced glucose metabolism (91, 92), and tolerised monocytes have increased mitochondrial respiratory activity (93). The bone marrow cells of LPS-tolerised mice have decreased TNF production (14) and macrophages exhibit increased IL-10 production (15). This change in cytokine profile may promote the induction of Treg cells (94). This is in keeping with the finding that after the induction of sepsis, tolerant animals had significantly and persistently higher numbers of Treg cells (95). β-glucan has been shown to reverse the epigenetic state of LPS-induced immunological tolerance (16, 91, 96). Trained immunity induced by BCG, adenovirus or β-glucan has been shown to promote MHC-II expression and TNF production, providing further support that these training molecules may be promising therapeutic interventions in sepsis patients and may help to restore both innate and adaptive immunoparalysis.

**Figure 2:**
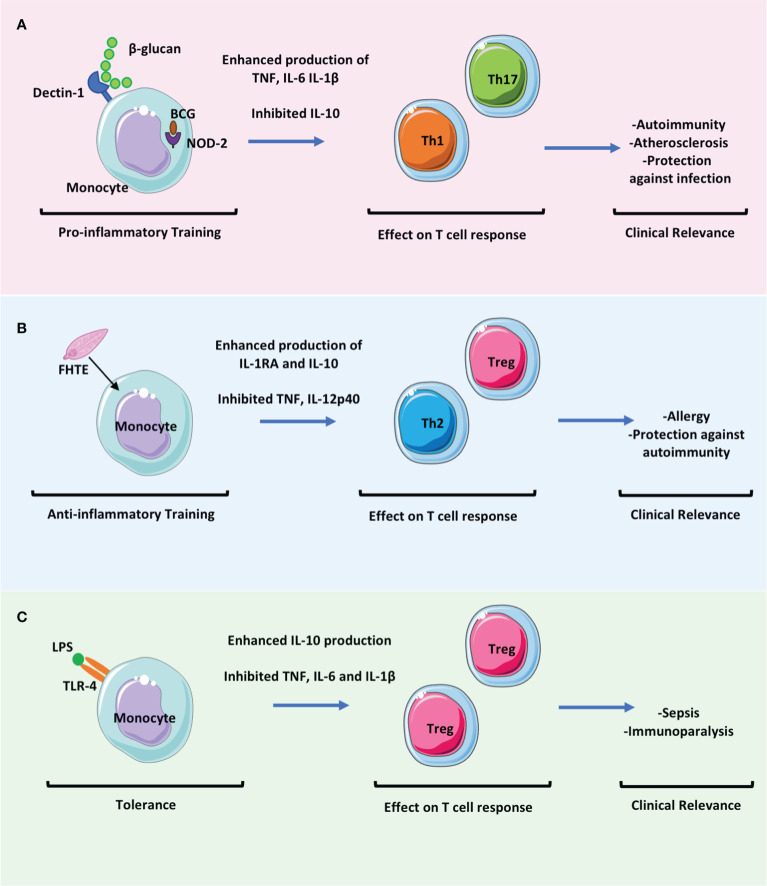
The effect of trained immunity on T cell responses will be key to determining the clinical relevance of trained immunity in mediating protection versus pathology. Trained immunity likely has an impact in many settings of infectious diseases and in immune mediated pathology. These clinically relevant effects may be mediated directly by the altered immune responses of myeloid cells but also indirectly by the effects of these myeloid cells on adaptive immune responses. **(A)** Trained immunity induced by β-glucan or BCG results in enhanced proinflammatory monocyte function which may promote the activation and differentiation of Th1 and Th17 cells whilst concomitantly downregulating Treg cell responses. This may be beneficial in promoting protection against infection but may be pathogenic in settings of immune mediated pathology such as in autoimmunity or atherosclerosis. **(B)** Fasciola hepatica total extract (FHTE) induces trained immunity which results in enhanced anti-inflammatory responses. This may be beneficial in attenuating autoimmune diseases but may promote cancer or allergy. **(C)** LPS induced trained immunity followed by restimulation with LPS results in tolerance. This induces Treg cells and is thought be pathological during sepsis.

In summary, defining the effect of trained immunity on T cell responses in discreet disease settings will be key to determining the clinical relevance of trained immunity in mediating protective or pathological immune responses. We acknowledge that identifying trained immunity and discriminating it from chronic inflammation or immune dysfunction is difficult in settings of clinically established disease. **Figure 2** summarises how different types of trained immunity may promote differential T cell responses and the disease context with which these are associated.

## Conclusions

In this review, we sought to examine the emerging evidence that trained immunity may affect and be affected by T cell responses. Recent studies have indicated that T cells play a role in modulating trained immunity and there is a clear rationale to support the hypothesis that innate immune training will affect adaptive responses *via* the modulation of innate proinflammatory cytokine production and APC function (**Table 1**). Knowledge gaps remain in understanding the impact of trained immunity on the adaptive immune response, both in settings of acute and chronic inflammation. More specifically, there is still limited information on how specific drivers of trained immunity may differentially affect subpopulations of T cells and disrupt the delicate Treg/Th17 or Th1/Th2 balance, for example. Conversely, understanding how certain populations of T cells regulate the induction of trained immunity may help to identify cell targets for therapeutic interventions. A direct cause and effect relationship between trained immunity and T cell responses remains to be determined, however, there is evidence from current literature, discussed in this review, to suggest that this relationship may exist, at least in the contexts of certain inflammatory conditions.

Although evidence is emerging to suggest that trained immunity may precipitate autoimmune, autoinflammatory and allergic diseases, BCG vaccination has been paradoxically associated with reduced risk of allergy and autoimmune disease (97–101). This raised the question of how trained immunity induced by different stimuli may result in divergent health or disease outcomes. Since T cells play a critical role in mediating pathological inflammation in these settings, the effects of trained immunity on the T cell response may be a key to determining how the balance falls in favour of inflammation versus resolution in different settings.

Since protective and deleterious nonspecific effects of vaccines are associated with sex (46, 102–105), sex differences may be another key factor in determining outcomes of trained immunity. Interestingly, in a recent trained immunity study on healthy adults, BCG vaccination was associated with significantly reduced inflammatory markers in males only (106). This is in keeping with observations that neonatal BCG vaccination elicits a strong protective effect against all-cause mortality early in boys but later in girls (104). This is also consistent with the evidence that pre-term male neonates are at higher risk of mortality compared with their female counterparts (107). This trend follows through life, with males being more likely to die from sepsis than females (108, 109), an observation associated with altered ratios of proinflammatory IL-6 and anti-inflammatory IL-10. Conversely, females are at increased risk of developing autoimmune disease, with a role for IFN-γ postulated to be central to this predisposition (110, 111). IFN-γ is produced by both innate and adaptive immune cells and plays a critical role in the induction of trained immunity (13, 25). Therefore, IFN-γ production may be a key determinant in inducing trained immunity in males and females. IFN-γ may also be a critical influence on the outcome of trained immunity; tipping the balance from protection to immune-mediated pathology.

The ability to induce trained immunity may change over a human lifetime. Since defects in immunity leave people uniquely susceptible to infections at the extremes of life, better understanding of how trained immunity can impact the immune response to infection in the very young and the very old will be critical to translating this knowledge towards the design of vaccines specifically for these vulnerable populations. It is noteworthy that the T cell repertoire is small in infancy and increases over time as lymphoid progenitor cells egress from the bone marrow and mature in the thymus. At the other extreme of the lifespan, the diversity of the T cell repertoire is reduced due to thymic involution and the predominance of clonally expanded populations due to previously encountered pathogens (112). This is thought to result in a shift to rely on innate immune cell function in the very young and the very old. Trained immunity has been implicated in processes driving ‘inflammageing’ and in age-associated neurodegenerative disease such as Alzheimer’s disease (2, 113). Since a skewing of haematopoiesis occurs during trained immunity (12, 25), resulting in increased ratios of myelopoiesis to lymphopoieses, it is worthwhile considering how this may affect the T cell repertoire in the long-term. Further work elucidating the kinetics of these skewed ratios over time and what it means for lymphoid cell function will be critical, especially in infants who are forming a T cell repertoire in the thymus from cells that egress from the bone marrow. Conversely, trained immunity may serve as a necessary protective measure in infants to bridge the gap until the T cell repertoire is established.

We postulate that better understanding of the impact of trained immunity on adaptive immune responses will help to determine who benefits from different forms of immune memory; when, how and why.

## Author Contributions

DM: conceptualisation, investigation, writing- original draft, and visualisation. KM: conceptualisation, writing- review and editing, and funding acquisition. SB: conceptualisation, investigation, writing -original draft, review and editing, and funding acquisition. All authors contributed to the article and approved the submitted version.

## Funding

HRB Emerging Investigator Award EIA-2019-010 (awarded to SB).

## Conflict of Interest

The authors declare that the research was conducted in the absence of any commercial or financial relationships that could be construed as a potential conflict of interest.

## Publisher’s Note

All claims expressed in this article are solely those of the authors and do not necessarily represent those of their affiliated organizations, or those of the publisher, the editors and the reviewers. Any product that may be evaluated in this article, or claim that may be made by its manufacturer, is not guaranteed or endorsed by the publisher.
